# Incidence, severity, aetiology and type of neck injury in men's amateur rugby union: a prospective cohort study

**DOI:** 10.1186/1746-1340-18-18

**Published:** 2010-07-01

**Authors:** Michael S Swain, Henry P Pollard, Rod Bonello

**Affiliations:** 1 Macquarie Injury Management Group (MIMG), Faculty of Science, Macquarie University, Sydney, Australia

## Abstract

**Background:**

There is a paucity of epidemiological data on neck injury in amateur rugby union populations. The objective of this study was to determine the incidence, severity, aetiology and type of neck injury in Australian men's amateur rugby union.

**Methods:**

Data was collected from a cohort of 262 participants from two Australian amateur men's rugby union clubs via a prospective cohort study design. A modified version of the Rugby Union Injury Report Form for Games and Training was used by the clubs physiotherapist or chiropractor in data collection.

**Results:**

The participants sustained 90 (eight recurrent) neck injuries. Exposure time was calculated at 31143.8 hours of play (12863.8 hours of match time and 18280 hours of training). Incidence of neck injury was 2.9 injuries/1000 player-hours (95%CI: 2.3, 3.6). As a consequence 69.3% neck injuries were minor, 17% mild, 6.8% moderate and 6.8% severe. Neck compression was the most frequent aetiology and was weakly associated with severity. Cervical facet injury was the most frequent neck injury type.

**Conclusions:**

This is the first prospective cohort study in an amateur men's rugby union population since the inception of professionalism that presents injury rate, severity, aetiology and injury type data for neck injury. Current epidemiological data should be sought when evaluating the risks associated with rugby union football.

## Background

Neck injury in Rugby Union (RU) has a potential for devastating consequences[1]. For every debilitating spinal cord injury there may be as many as ten near misses[2,3]. Long term health implications, such as acquired degenerative change, have been reported from repetitive traumatic forces to the neck in RU [4,5].

The scientific process of preventing sports injury requires accurate and reliable understanding of the sports injury problem[6]. This initially surmounts to identifying the probability and consequence of the sports injury problem[7]. Subsequently aetiology and risk factors of the sports injury problem are then identified. With this knowledge the sequence of events which leads to sports injury can be objectively described and risk mitigation processes can be informed[8].

It is estimated neck injury accounts for between 3.5%[9] and 9.0%[10] of total injuries sustained in men's amateur RU. Only a small number of prospective cohort studies provide comparable inter-study definition on neck injury incidence and type in RU, albeit they are in either junior or professional populations[11-16]. There is a paucity of neck injury incidence, severity, aetiology and type data from amateur men's RU populations. This is particularly notable since the 1995 inception of professionalism in RU[17]. Amateur men are thought to comprise a large proportion of the 3 million strong rugby playing community[18].

The objective of this report was to present data on the incidence rate, severity, aetiology and type of neck injury in a cohort of Australian men's amateur RU playing population.

## Methods

Ethics approval was granted from the ethics review committee (human research), Macquarie University, Sydney, Australia (reference number: HE24FEB2006-M04460). Written approval was granted by senior club representatives and sports medicine personnel to conduct the study. Players gave written consent to participate in the study.

A prospective cohort study design was applied through the 2006 and 2007 rugby union seasons. Participants were recruited from two Australian RU clubs located in Sydney's northern suburbs. All participants recruited played in senior grades and were male aged 18 years or over (mean age: 24.1 years ± 5.7 years). Participants were recruited pre-season. Data collection and player monitoring was completed by the rugby clubs' sports medicine personnel after a training period to standardise all assessments and recording methods. The inclusion criterion for the club medical personnel was: a relevant tertiary health related qualification such as doctor, physiotherapist or chiropractor. Data collectors attended all training sessions and matches in an attempt not to miss the injuries of interest during the observation period.

Neck injury definition was all encompassing[19]. Neck injury was defined as any injury to the neck region which was sustained as a result of participation in rugby union which caused a reduction in the amount or level of sports activity, or need for advice or treatment, or adverse social or economic effects[7,20]. A visual pain diagram supplied in the data collection questionnaire oriented data collectors as to the region inclusive for neck injury. The data collection questionnaire was a modified version of the Rugby Union Injury Report Form for Games and Training (RUIRF)[21]. It was modified to collect specific details of neck injury such as symptoms of neck injury, visual range of motion findings, other physical orthopedic findings plus techniques, modalities and advice used in the management of neck injury. Details on mechanism of injury were gathered by data collectors through athlete interview immediately following the inciting injury event. The RUIRF includes the Orchard Sports Injury Classification System (version 8),[22] further adding details of injury type to collected data. The injury diagnosis was made by the clubs medical personal (data collectors) based on clinical examination findings. Incidence was reported as the number of neck injuries per 1000 player-hours. Attempts were made to measure actual exposure[7] time by including training time in exposure time. The formula: Incidence = 1000 × (number of neck injuries per season)/(1.33 games + trainings hours) (number of participants) was used in the calculation of incidence. Severity of injury was reported as the total number of weeks missed from play[23,24]. Severity was arbitrarily grouped as minor (less than one week lost from play), mild (1-2 weeks lost from play), moderate (2-3 weeks lost from play) and severe (3 + weeks lost from play).

Analysis of game versus training risk and injury rate required fitting a Poisson regression model. This analysis was undertaken using the statistical package GenStat. Associations between outcome measures and player position, phase of play, aetiology and injury type have been described by means of cross tabulation. Associations with injury severity were mostly conducted using ordinal logistic regression models. These analyses were undertaken using the statistical package Minitab. *P *< 0.05 was considered statistically significant, although values in the range 0.05 <*P *< 0.10 are worth commenting on for potential associations. Where appropriate, 95% confidence intervals (95%CI) were calculated. For incidence, these have been based on Poisson and binomial distribution assumptions for incidence and percentage results respectively. They were obtained using the standard errors of parameter estimates from GenStat's generalised linear model procedure.

## Results

The cohort consisted of 262 participants who were recruited over two seasons. A total of 90 (eight recurrent) neck injuries were recorded which affected 74 players. Exposure time for the cohort was calculated at 12863.8 hours of match time and 18280 hours of training totalling 31143.8 hours of play. Incidence of neck injury in this cohort was calculated to be 2.9 injuries/1000 player-hours (95%CI: 2.3, 3.6) with a recurrence incidence of 0.26 repeat injuries/1000 player-hours (95%CI: 0.13, 0.52). Of the neck injuries requiring medical attention on field, 46.5% resulted in the player retiring injured from play. The odds ratio for retiring versus return to play as a risk factor of injury severity was 7.01 (95%CI: 2.31, 21.29). Therefore players who retired injured were 7.01 times more likely to have a more time off compared to a player who did return to play. In regards to time lost from play 69.3% of neck injuries required no additional weeks off from play, 17% missed one additional week of play, 6.8% of injured players missed two weeks from play and 6.8% of players missed three or more weeks from play. Two neck injuries were unable to be tracked and had unknown severity. As expected players who returned to play tended to have far less subsequent time off play (*P *= 0.000). A single spinous process avulsion fracture not affecting the lamina loosely matched the definition of serious cervical spine injury during this study. No fatal or non-fatal catastrophic injuries were reported during the study period.

### Game versus training

Game injuries at 85.6% (N = 77) of total neck injuries were more frequent than training injuries which accounted for 14.4% of neck injuries. The incidence of neck injury due to match play was 5.99 injuries/1000 player-hours (95%CI: 4.77, 7.52) while training incidence was 0.71 injuries/1000 player-hours (95%CI: 0.41, 1.24). The incidence of neck injury was significantly higher in games than in training (*P *< 0.001), with the risk being 8.4 times greater (95%CI: 4.6, 15.3). A similar severity pattern was apparent between game and training neck injuries (Table 1). There was no detectable association between game versus training and neck injury severity (P = 0.50).

**Table 1:** Incidence and severity of game and training neck injuries

Severity	Game	Training	All
**Minor**	N = 51	N = 10	N = 61
	3.96 (CI: 2.95, 5.21)	0.55 (CI: 0.26, 1.01)	1.96 (CI: 1.50, 2.52)
	68.0%	76.9%	

**Mild**	N = 13	N = 2	N = 15
	1.01 (CI: 0.54, 1.73)	0.11 (CI: 0.01, 0.40)	0.48 (CI: 0.27, 0.79)
	17.3%	15.4%	

**Moderate**	N = 6	N = 0	N = 6
	0.47 (CI: 0.17, 1.02)	0.00	0.19 (CI: 0.07, 0.42)
	8.0%	0.0%	

**Severe**	N = 5	N = 1	N = 6
	0.39 (CI: 0.13, 0.91)	0.06 (CI: 0.00, 0.30)	0.19 (CI: 0.07, 0.42)
	6.7%	7.7%	

Game incidence/1000 game player-hours (95% CI)

Training incidence/1000 training player-hours (95% CI)

All incidence/1000 player-hours (95% CI)

### Position

The hooker, front row and back row positions demonstrated the highest number of neck injuries in this cohort, while the fullback and wingers followed by the five-eighth and midfield backs demonstrated the lowest number of neck injuries (Table 2). Whilst only forwards fell into the category of the most severe injuries (3 + weeks lost from play), there was no detectable association between position of play and neck injury severity (*P *= 0.88) (Table 3).

**Table 2:** Incidence of neck injury as a factor of player position

Position of play	Count (N)	Incidence/1000 player hours	Percent %
**Forwards**	(71)	4.27 (CI: 3.34, 5.39)	78.9%

**Back Row**	(25)	4.01 (CI: 2.60, 5.92)	27.8%

**LF**	(8)	3.85 (CI: 1.66, 7.59)	8.9%

**8**	(8)	3.85 (CI: 1.66, 7.59)	8.9%

**RF**	(9)	4.33 (CI: 1.98, 8.23)	10.0%

**Second Row**	(12)	2.89 (CI: 1.49, 5.05)	13.3%

**LL**	(8)	3.85 (CI: 1.66, 7.59)	8.9%

**RL**	(4)	1.93 (CI: 0.52, 4.93)	4.4%

**Front Row**	(34)	5.46 (CI: 3.78, 7.63)	37.8%

**LHP**	(9)	4.33 (CI: 1.98, 8.23)	10.0%

**H**	(15)	7.22 (CI: 4.04, 11.92)	16.7%

**THP**	(10)	4.82 (CI: 2.31, 8.86)	11.1%

**Backs**	(19)	1.31 (CI: 0.79, 2.04)	21.1%

**Inside Backs**	(6)	1.44 (CI: 0.53, 3.14)	6.7%

**IC**	(3)	1.44 (CI: 0.30, 4.22)	3.3%

**OC**	(3)	1.44 (CI: 0.30, 4.22)	3.3%

**Outside Backs**	(4)	0.64 (CI: 0.17, 1.64)	4.4%

**LW**	(1)	0.48 (CI: 0.01, 2.68)	1.1%

**RW**	(2)	0.96 (CI: 0.12, 3.48)	2.2%

**FB**	(1)	0.48 (CI: 0.01, 2.68)	1.1%

**Scrum Halves**	(9)	2.17 (CI: 0.99, 4.11)	10.0%

**HB**	(7)	3.37 (CI: 1.36, 6.95)	7.8%

**5/8**	(2)	0.96 (CI: 0.12, 3.48)	2.2%


**Table 3:** Severity of neck injury as a factor of grouped player position

Severity	Back Row (3)	Front Row (3)	Inside Backs (2)	Outside Backs (3)	Scrum Halves (2)	Second Row (2)	Backs (7)	Forwards (8)
**Minor**	N = 17	N = 22	N = 4	N = 2	N = 8	N = 8	N = 14	N = 47
	2.73 (CI: 1.59, 4.37)	3.53 (CI: 2.21, 5.35)	0.96 (CI: 0.26, 2.47)	0.32 (CI: 0.04, 1.16)	1.93 (CI: 0.83, 3.80)	1.93 (CI: 0.83, 3.80)	0.96 (CI: 0.53, 1.62)	2.83 (CI: 2.08, 2.76)
	70.8%	64.7%	66.7%	66.7%	88.9%	66.7%	77.8%	67.1%

**Mild**	N = 6	N = 5	N = 0	N = 1	N = 1	N = 2	N = 2	N = 13
	0.96 (CI: 0.35, 2.10)	0.80 (CI: 0.26, 1.87)	0.00	0.16 (CI: 0.00, 0.89)	0.16 (CI: 0.00, 0.89)	0.48 (CI: 0.06, 1.74)	0.14 (CI: 0.02, 0.50)	0.78 (CI: 0.42, 1.34)
	25.0%	14.7%	0.0%	33.3%	11.1%	16.7%	11.1%	18.6%

**Moderate**	N = 0	N = 3	N = 2	N = 0	N = 0	N = 1	N = 2	N = 4
	0.00	0.48 (CI: 0.10, 1.41)	0.48 (CI: 0.06, 1.74)	0.00	0.00	0.16 (CI: 0.00, 0.89)	0.14 (CI: 0.02, 0.50)	0.24 (CI: 0.07, 0.62)
	0.0%	8.8%	33.3%	0.0%	0.0%	8.3%	11.1%	5.7%

**Severe**	N = 1	N = 4	N = 0	N = 0	N = 0	N = 1	N = 0	N = 6
	0.16 (CI: 0.00, 0.89)	0.62 (CI: 0.17, 1.64)	0.00	0.00	0.00	0.16 (CI: 0.00, 0.89)	0.00	0.36 (CI: 0.13, 0.79)
	4.2%	11.7%	0.0	0.0%	0.0%	8.3%	0.0%	8.6%

Neck injury count

Incidence/1000 player-hours (95% CI)

Percent

Further analysis was performed on groups of player positions. In this cohort 78.9% of neck injuries affected the forwards and 21.1% of injuries were sustained by backs. There was a high proportion of injuries in the forward positions. From the cross tabulation, there was no apparent difference in forward versus back position and neck injury severity (Table 3). This was further supported by the ordinal logistic regression analysis, which confirms there is no difference in time off for those that are injured and forward versus back position (*P *= 0.36). Further tabulation of player position into groups of front row, second row, back row, scrum halves, inside backs and outside backs was included (Table 2). There was an uneven distribution of injuries across this grouping with an apparent excess in the back and front row positions. Again, the expected number can be calculated in proportion to the number of player positions within each grouping. This analysis confirms the significant differences in injury frequency across the player positions (*P *= 0.000). The contribution to the chi-square statistic indicates the excess of the front row injuries, but also the relatively low rate for the outside backs (wingers and fullback). However, there was no significant difference in injury severity across this grouping, as indicted by the cross tabulation, and the results of an ordinal logistic regression analysis (*P *= 0.68). Finally comparison was made between injury frequency of front row and back row players. In this cohort there was no significant difference in the neck injury frequency of back row and front row (*P *= 0.30). Consequently, there was no significant difference in neck injury severity between these two groups (*P *= 0.42).

### Phase of play

The tackle phase of play demonstrated the greatest number of neck injuries in this cohort followed by the scrum and ruck (Table 4). The tackle phase and scrum demonstrated the most severe (3 + weeks lost from play) neck injuries however, there was no detectable association between phase of play and neck injury severity (*P *= 0.27) (Table 5).

**Table 4:** Incidence of neck injury as a factor of phase of play and player position

Phase of play	Back Row	Front Row	Inside Back	Outside Back	Scrum Halves	Second Row	All
**Collision**	N = 0	N = 0	N = 1	N = 0	N = 0	N = 0	N = 1
	0.00	0.00	0.24 (CI: 0.01, 1.34)	0.00	0.00	0.00	0.03 (CI: 0.00, 0.18)
	0.0%	0.0%	16.7%	0.0%	0.0%	0.0%	1.2%

**Lineout **	N = 0	N = 0	N = 0	N = 0	N = 0	N = 1	N = 1
	0.00	0.00	0.00	0.00	0.00	0.16 (CI: 0.00, 0.89)	0.03 (CI: 0.00, 0.18)
	0.0%	0.0%	0.0%	0.0%	0.0%	8.3%	1.2%

**Maul**	N = 2	N = 2	N = 0	N = 0	N = 0	N = 2	N = 6
	0.32 (CI: 0.04, 1.16)	0.32 (CI: 0.04, 1.16)	0.00	0.00	0.00	0.48 (CI: 0.06, 1.74)	0.19 (CI: 0.07, 0.42)
	8.3%	6.3%	0.0%	0.0%	0.0%	16.7%	6.9%

**Ruck**	N = 5	N = 5	N = 1	N = 1	N = 4	N = 4	N = 20
	0.80 (CI: 0.26, 1.87)	0.80 (CI: 0.26, 1.87)	0.24 (CI: 0.01, 1.34)	0.16 (CI: 0.00, 0.89)	0.96 (CI: 0.26, 2.47)	0.96 (CI: 0.26, 2.47)	0.64 (CI: 0.32, 0.99)
	20.8%	15.6%	16.7%	25.0%	44.4%	33.3%	23.0%

**Scrum**	N = 2	N = 20	N = 0	N = 0	N = 0	N = 0	N = 22
	0.32 (CI: 0.04, 1.16)	3.21 (CI: 1.96, 4.96)	0.00	0.00	0.00	0.00	0.71 (CI: 0.44, 1.07)
	8.3%	62.5%	0.0%	0.0%	0.0%	0.0%	25.3%

**Tackle**	N = 15	N = 5	N = 4	N = 3	N = 5	N = 5	N = 37
	2.41 (CI: 1.35, 3.97)	0.80 (CI: 0.26, 1.87)	0.96 (CI: 0.26, 2.47)	0.48 (CI: 0.10, 1.04	1.20 (CI: 0.39, 2.81)	1.20 (CI: 0.39, 2.81)	1.19 (CI: 0.84, 1.64)
	62.5%	15.6%	66.7%	75.0%	55.6%	41.7%	42.5%

Neck injury count

Incidence/1000 player-hours (95% CI)

Percent

**Table 5:** Severity (count) of neck injury as a factor of phase of play

Severity	Collision	Lineout	Maul	Ruck	Scrum	Tackle
**Minor**	N = 1	N = 1	N = 5	N = 17	N = 13	N = 23
	0.03 (CI: 0.00. 0.18)	0.03 (CI: 0.00. 0.18)	0.16 (CI: 0.05, 0.37)	0.55 (CI: 0.31, 0.87)	0.42 (CI: 0.22, 0.71)	0.74 (CI: 0.47, 1.11)
	100.0%	100.0%	83.3%	85.0%	59.1%	65.7%

**Mild **	N = 0	N = 0	N = 0	N = 2	N = 3	N = 8
	0.00	0.00	0.00	0.02 (CI: 0.01, 0.23)	0.10 (CI: 0.02, 0.28)	0.26 (CI: 0.11, 0.51)
	0.0%	0.0%	0.0%	10.0%	13.6%	22.9%

**Moderate**	N = 0	N = 0	N = 1	N = 1	N = 3	N = 1
	0.00	0.00	0.03 (CI: 0.00. 0.18)	0.03 (CI: 0.00. 0.18)	0.10 (CI: 0.02, 0.28)	0.03 (CI: 0.00. 0.18)
	0.0%	0.0%	16.7%	5.0%	13.6%	2.9%

**Severe**	N = 0	N = 0	N = 0	N = 0	N = 3	N = 3
	0.00	0.00	0.00	0.00	0.10 (CI: 0.02, 0.28)	0.10 (CI: 0.02, 0.28)
	0.0%	0.0%	0.0%	0.0%	13.6%	8.6%

Neck injury count

Incidence/1000 player-hours (95% CI)

Percent

Further analysis compared phase of play with player position for correlation. However, the counts were too low to make meaningful tests of associations. To overcome this, some of the less frequent categories were removed, namely collision, lineout, maul (phase of play) and inside backs, outside backs and scrum halves (player position). The reduced table then facilitated a chi-square test of association. The overall level of significance for association is *P *= 0.000, indicating a strong association. Comparing the observed and expected frequencies, it is evident that in the back row, there is an excess of tackle injuries, and a deficit of scrum injuries, whereas in the front row, this pattern is reversed. With regards to injury severity, it was found through tabulation of ordinal logistic regression that neither position nor phase of play influence injury severity (*P *= 0.30).

### Mechanism of injury

There were up to four injury mechanisms listed per neck injury suggesting force directions that cause neck injury are seldom uniplanar. The following table shows the absolute numbers, and also as a percentage (relative to the total number of injuries, i.e. 90) (Table 6).

**Table 6:** Aetiology and incidence of neck injury

Mechanism	Count	%
**Compression**	49	54%

**Flexion**	38	42%

**Rotation**	20	22%

**Side bend**	31	34%

**Unknown**	6	7%

As multiple mechanisms of injury were recorded the mechanism was coded into the presence/absence of each of the mechanisms; compression, extension, flexion, rotation, and side bend. A separate analysis was then undertaken for each of these mechanisms. The following table shows the number that reported each specific mechanism, shown for each 'severity' group (Table 7).

**Table 7:** Aetiology and severity of neck injury

Severity	Compression	Extension	Flexion	Rotation	Side bend	All
**Minor**	32	7	27	13	21	58

**Mild**	12	1	5	2	6	13

**Moderate**	4	1	2	1	3	5

**Severe**	3	1	4	3	1	6

**All**	51	10	38	19	31	82

Note that the sum of the counts will exceed the 'All' column, due to multiple mechanisms reported per injured player.

Neck injury count

There is some evidence of a weak association between time lost and presence of a compression mechanism injury (*P *= 0.073), with more time lost when this mechanism occurs, compared to when it did not. The odds ratio for compression as a risk factor was 2.62 (95% CI: 0.89-7.73) therefore players reporting this injury mechanism are 2.62 times more likely to have time lost from play compared with a player who did not report this mechanism. No other mechanism was associated with injury severity (all *P *> 0.5).

### Orchard Sports Injury Classification (OSICS-8)

Cervical facet joint injury was the most frequently recorded (42%) classification of neck injury, followed by brachial plexus/cervical nerve root injury (stinger/burner). At face value these injuries appeared to be associated with the highest time lost from play (Table 8). However, more formal analysis revealed no detectable association between OSICS-8 and neck injury severity (*P *= 0.35). Further comparison was made between Orchard sports injury classification with phase of play. The overall cross tabulation between phase of play grouping and OSICS-8 (Table 9) indicates some low frequencies, preventing an overall analysis of association. However, a sub set of data involving only brachial plexus/cervical nerve root injury (stinger/burner) and cervical facet joint injury, as well as scrum and tackle was extracted. The association of these low-frequency data was analysed using a Fisher's exact test for a 2 × 2 table. There was no significant relationship between tackle versus scrum and injury type (NP1 versus SN1) (*P *= 0.22). Further on relationship with severity was examined via an ordinal logistic regression to assess the effect of both Orchard sports injury classification (NP1 versus SN1) and phase of play (Scrum versus Tackle). No significant effect of either of these terms on injury severity (*P *= 0.30) was identified in this cohort.

**Table 8:** Injury type incidence and severity

Severity	NG1	NJ1	NL1	NM1	NN1	NP1	NZ1	SN1	NP1 SN1
**Minor**	N = 0	N = 1	N = 7	N = 11	N = 1	N = 27	N = 8	N = 6	
	0.00	0.03 (CI: 0.00, 0.18)	0.22 (CI: 0.09, 0.46)	0.35 (CI: 0.18, 0.63)	0.03 (CI: 0.00, 0.18)	0.87 (CI: 0.57, 1.26)	0.26 (CI: 0.11, 0.51)	0.19 (CI: 0.07, 0.42)	
	0.0%	100.0%	58.3%	84.6%	50.0%	71.1%	88.9%	46.2%	

**Mild**	N = 1	N = 0	N = 3	N = 1	N = 1	N = 6	N = 0	N = 3	
	0.03 (CI: 0.00, 0.18)	0.00	0.10 (CI: 0.02, 0.28)	0.03 (CI: 0.00, 0.18)	0.03 (CI: 0.00, 0.18)	0.19 (CI: 0.07, 0.42)	0.00	0.10 (CI: 0.02, 0.28)	
	100.0%	0.0%	25.0%	7.7%	50.0%	15.8%	0.0%	23.1%	

**Moderate**	N = 0	N = 0	N = 1	N = 1	N = 0	N = 2	N = 1	N = 1	
	0.00	0.00	0.03 (CI: 0.00, 0.18)	0.03 (CI: 0.00, 0.18)	0.00	0.06 (CI: 0.01, 0.23)	0.03 (CI: 0.00, 0.18)	0.03 (CI: 0.00, 0.18)	
	0.0%	0.0%	8.3%	7.7%	0.0%	5.3%	11.1%	7.7%	

**Severe**	N = 0	N = 0	N = 1	N = 0	N = 0	N = 3	N = 0	N = 2	
	0.00	0.00	0.03 (CI: 0.00, 0.18)	0.00	0.00	0.10 (CI: 0.02, 0.28)	0.00	0.06 (CI: 0.01, 0.23)	
	0.0%	0.0%	8.3%	0.0%	0.0%	7.9%	0.0%	13.4%	

**All**	N = 1	N = 1	N = 12(1)	N = 13	N = 2(2)	N = 38(3)	N = 9	N = 13*(2)	N = 1*
	0.03 (CI: 0.00, 0.18)	0.03 (CI: 0.00, 0.18)	0.38 (CI: 0.20, 0.67)	0.42 (CI: 0.22, 0.71)	0.06 (CI: 0.01, 0.23)	1.22 (CI: 0.86, 1.67)	0.28 (CI: 0.13, 0.55)	0.42 (CI: 0.22, 0.71)	0.03 (CI: 0.00, 0.18)
	1.1%	1.1%	13.3%	13.3%	2.2%	42.2%	10.0%	14.4%	1.1%

*Two injuries with unknown severity

INJURY TYPE ABBREVIATION

NG1: Avulsion fracture (spinous process) of the cervical spine, NJ1: Whiplash/neck sprain, NL1: Neck ligament injury, NM1: Neck muscle strain, NM1 (Contusion): Neck muscle contusion, NN1: Cervical nerve root compression/stretch, NP1: Cervical facet joint pain, NZ1: Neck pain undiagnosed, SN1: Brachial plexus traction injury/stinger/burner

Neck injury count (recurrent neck injury count)

Incidence/1000 player-hours (95% CI)

Percent

**Table 9:** Injury type count as a factor of phase of play

Injury type	Ruck	Scrum	Tackle
**NG1**	N = 0	N = 0	N = 0
	0.00	0.00	0.00
	0.0%	0.0%	0.0%

**NJ1**	N = 0	N = 0	N = 0
	0.00	0.00	0.00
	0.0%	0.0%	0.0%

**NL1**	N = 3	N = 5	N = 4
	0.10 (CI: 0.02, 0.28)	0.16 (CI: 0.05, 0.37)	0.13 (CI: 0.03, 0.33)
	15.0%	22.7%	10.8%

**NM1**	N = 3	N = 4	N = 2
	0.10 (CI: 0.02, 0.28)	0.13 (CI: 0.03, 0.33)	0.06 (CI: 0.01, 0.23)
	15.0%	18.2%	5.4%

**NN1**	N = 0	N = 0	N = 2
	0.00	0.00	0.06 (CI: 0.01, 0.23)
	0.0%	0.0%	5.4%

**NP1**	N = 11	N = 9	N = 17
	0.35 (CI: 0.18, 0.63)	0.29 (CI: 0.13, 0.55)	0.55 (CI: 0.32, 0.87)
	55.0%	40.9%	45.9%

**NZ1**	N = 2	N = 3	N = 2
	0.06 (CI: 0.01, 0.23)	0.10 (CI: 0.02, 0.28)	0.06 (CI: 0.01, 0.23)
	10.0%	13.6%	5.4%

**SN1**	N = 1	N = 1	N = 10
	0.03 (CI: 0.00, 0.18)	0.03 (CI: 0.00, 0.18)	0.32 (CI: 0.15, 0.59)
	5.0%	4.5%	27.0%

Injury type abbreviation

NG1: Avulsion fracture (spinous process) of the cervical spine, NJ1: Whiplash/neck sprain, NL1: Neck ligament injury, NM1: Neck muscle strain, NN1: Cervical nerve root compression/stretch, NP1: Cervical facet joint pain, NZ1: Neck pain undiagnosed, SN1: Brachial plexus traction injury/stinger/burner

Neck injury count

Incidence/1000 player-hours (95% CI)

Percent

## Discussion

To the authors knowledge this is the first prospective study of neck injury in an amateur men's population since the inception of the professional RU era. Via an all inclusive injury definition and calculation of game, training and overall parameters of exposure time, incidence of neck injury in an amateur RU cohort is estimated as: 5.99/1000 match player-hours, 0.71/1000 training player-hours and 2.9/1000 play-hours. Furthermore, a minimum of 50 player weeks was lost from play. An intuitive pattern of neck injury resulted: less severe injuries occurred most frequently and most severe neck injuries occurred least frequently. Aetiology of neck injury in this study was seldom found to be a result of uniplanar neck movement, as several planes of movement were commonly reported per neck injury. The most frequently occurring neck injury type in this population was cervical facet joint injury as assessed by tertiary qualified data collectors.

There are limitations in studying amateur sporting populations, which may not be as apparent in the professional arena. It is important to reliably identify athletic exposure[25]. In this study population the position of play sometimes varied throughout the season. For example, front row players sometimes played games in the centre position, which limits the accuracy of incidence by position of play. As such cautious estimation has been reported on player position data. Methods of assessing mechanism of injury and sports injury type pose a challenge in sports injury epidemiology. The ability of injured athletes to comprehend and recall what actually took place when they were injured is debateable, and a limitation of this study. This is due to the speed at which injury events occur and the propensity for neck injury to be associated with head injury and disorientation[26]. Furthermore the ability of a clinician to describe a tissue injury diagnosis through subjective examination is limited[27]. In this study more objective criteria such as ultrasound and magnetic resonance imaging were precluded due to costs and practicality.

Paucity in the literature of similar population with similar injury definition and study design limits comparison of these results with parallel studies. Since the commencement of this study consensus has been achieved on injury definitions and data collection procedures for studies of injuries in RU[28]. This is crucial for meaningful comparison of studies in the future.

Recent estimation of match play neck injury incidence in professional RU populations was reported to range between 4.2 (95%CI: 2.1, 8.3) and 6.46 (95%CI: 5.31, 7.86)/1000 player-hours[29,30]. The incidence of neck injury in this study fell within this range of professional RU reports. This finding appears to conflict with the trend noted elsewhere,[18] that increasing injury incidence is related with higher levels of play. Comparison of studies with disparate injury definitions must be undertaken with caution. Further studies on amateur populations are required to identify if neck injury incidence in amateurs mirrors that of professional RU populations.

This and other prospective cohort studies in RU[29-33] have found a higher incidence of neck injury match play compared to training. Similar to all studies is the greater exposure time to training than match play. Brooks et al[33] considers the contact phases of training very high risk while non-contact phase of training to be very low risk. Suggested reasons for a high injury rate of match play injury are associated with game intensity and player fatigue,[14] they include; 1) Increased 'ball in play' time in match situations, as seen in the professional era, therefore increased exposure to contact situations;[32] 2) In part training focus may change from skills to conditioning;[32] 3) There may have been a de-emphasis of the contact phase of skill training[32]. Further to injury rate, severity of game versus training neck injury appears similar to other studies[29-33] with a similar average[29] severity.

Recent evaluation of the tackle phase of rugby union suggest ball carriers[34,35] and tacklers[34] are at risk of head and neck injury. This study found the back row players to be particularly susceptible to tackle related neck injury. Indeed data observed by Quarrie and Hopkins[35] and Fuller et al[34] suggest the back row players have a high exposure to tackle events which may be reflected in this study's findings. In addition, this and other[36-38] studies found neck injury occurred in the forwards more frequently than the backs. The front row players, namely the hookers, were most frequently affected by neck injury through their role in the scrum phase of play. The high risk component of this phase is engagement and subsequent collapse as a result of improper engagement[1]. Ongoing vigilance towards player safety is required in scrums.

The tackle phase of play in this study was found to demonstrate the greatest number of neck injuries, followed by the scrum and ruck. A similar trend has been identified elsewhere for all injury types in amateur RU[9,33,39]. Recently Fuller et al[40] commented on the relative propensity for contact events to cause injury in RU. This was achieved by adjusting the injury probability to represent the contact event exposure time. They found relative to exposure, collisions were 70% more likely and scrums 60% more likely to result in an injury than a tackle[40]. Such adjustment for exposure time per contact event was not accounted for in this study and acknowledged as a limitation.

Compression was found to be the most frequently described mechanism of neck injury in amateur men, followed by flexion then side-bend of the neck. Indeed these results support the concept of Winkelstein and Myers,[41] who suggest uni-planar compression force is too simplistic. Compressive and shear forces are generated during a tackle situation by the combined effort of neck, head and shoulder areas[42]. Cervical compression via a blow to the vertex of the head has previously been identified as a high risk mechanism associated with scrum impact[43] and tackles[44,45]. When neck compression was a factor in this study, the severity of neck injury appeared to be greater. Indeed, more sinister injuries in athletes such as burst fracture, fracture dislocation, lamina fracture and collapsed vertebra have been associated with the mechanism of axial compression with or without rotation or hyper-flexion[46].

The OSCIS-8 is considered the preferred coding system for sports medicine research[47] which has more recently been superseded by a 10^th ^version[48]. Facet joint and nerve root/brachial plexus injuries have been previously identified as the most common neck injury type in professional RU[29] as in this study. Conversely, no significant correlation was found between type of neck injury and severity in this study. Additionally correlations between phases of play and neck injury type, could not be found. Therefore disparity exists between the findings of Fuller et al[29] that scrummaging had a higher frequency of facet mediated problems and the tackle phase had more cervical nerve root injury type[29]. A larger data pool may identify a relationship between phase of play and neck injury type in an amateur rugby union population.

## Conclusions

Severe neck injuries still occasionally occur in RU, particularly at the amateur level[49]. Stakeholders such as coaches, policy makers and sports medicine personnel should seek epidemiological data when evaluating the risk associated with the practice of RU football. Sound prevention and management strategies targeted at neck injury in RU require current information obtained through best available methods. The results of this study provide a yardstick for the incidence, severity, aetiology and type for future neck injury surveillance in Australian men's amateur RU.

## Competing interests

No funding was received in the preparation of this manuscript. The authors have no conflict of interest directly related to the content of the manuscript.

## Authors' contributions

MSS conceived the study, participated in the study design, collected baseline data, trained data collector, collected data from games and trainings of LRU, drafted and edited the manuscript. HPP conceived the study, participated in the study design, helped to draft and edit the manuscript. RB conceived the study, participated in the study design, helped to draft and edit the manuscript. All authors read and approved the manuscript.
